# Quantity Does Not Matter: Number, Ratio, or Grouping of Hodgkin/Reed–Sternberg Cells Does Not Affect Prognosis in Patients with Classic Hodgkin Lymphoma

**DOI:** 10.3390/medicina62081569

**Published:** 2026-08-17

**Authors:** Burcin Pehlivanoglu, Nazimcan Tezel, Osman Can Ozturk, Serra Begum Emecen, Mehmet Ali Ozcan, Sermin Ozkal

**Affiliations:** 1 Department of Pathology, Faculty of Medicine, Dokuz Eylul University, Balcova, Izmir 35330, Turkey; 2 Department of Molecular Pathology, Insitute of Health Sciences, Dokuz Eylul University, Balcova, Izmir 35330, Turkey; 3 Department of Hematology, Faculty of Medicine, Dokuz Eylul University, Balcova, Izmir 35330, Turkey

**Keywords:** classic Hodgkin lymphoma, neoplastic cells, prognosis, CD30, Hodgkin cells, Reed–Sternberg cells

## Abstract

*Background and Objectives:* The prognostic effect of the number of neoplastic cells in classic Hodgkin lymphoma (CHL) regardless of the histological subgroup has not been studied to date. We aimed to evaluate the prognostic impact of the number, ratio, and/or grouping of Hodgkin/Reed–Sternberg (HRS) cells in patients with CHL. *Materials and Methods:* 135 consecutive adult patients with CHL were included. The number, ratio, and grouping of HRS cells were evaluated on hematoxylin–eosin (HE) and CD30-stained slides. The ratio and group formation were scored. *Results:* Female:male ratio was 0.82. The median age was 36 ± 15.8 (range: 18–82 years). The number of HRS cells was significantly higher in nodular sclerosis CHL than in mixed-cellularity CHL and lymphocyte-rich CHL. Also, the number of HRS cells was significantly higher in syncytial variant nodular sclerosis CHL (SV-NSCHL). HRS cell ratio was in the range of 6–25% in more than 50% of the cases on both HE- and CD30-stained slides. The number of cases with a group score of 0 (no HRS groups) was significantly higher among men compared to women. HRS cells mostly did not form groups in EBER-positive patients. Confluent/large groups were more common in SV-NSCHL. Response to first-line therapy, gender, and EBER positivity were found to be independent prognostic factors. The number, ratio, and grouping of HRS cells were not associated with OS, stage, recurrence, and/or response to first-line treatment. *Conclusions:* The number, ratio, and/or grouping of HRS cells do not have any significant prognostic impact on CHL patients, however future studies are required to explore their pathogenetic implications.

## 1. Introduction

Classic Hodgkin lymphoma (CHL) is a relatively rare lymphoid neoplasm of abnormal (defective) germinal-center B cells and has four subgroups: (1) nodular sclerosis, (2) lymphocyte-rich, (3) mixed cellularity, and (4) lymphocyte-depleted [1]. These subgroups show some morphological differences. While the CHL subgroup was initially considered to affect prognosis, histologic subtype does not seem to have a significant prognostic effect following the implementation of multimodal therapy strategies into clinical practice, especially in early-stage patients [2].

Tumor size is a major prognostic factor for several types of solid tumors. Even debulking surgeries are performed to get better survival outcomes. For lymphomas, however, the disease is staged based on the extent of the disease, rather than the tumor size, and the number of neoplastic cells is of less clinical significance. While this is also the case for CHL, CHL is somehow unique as it is usually characterized by a very small number of neoplastic cells. In the majority of cases, large mononuclear Hodgkin (H) cells and/or multinuclear Reed–Sternberg (RS) cells with prominent eosinophilic nuclei are scattered among non-neoplastic inflammatory cells, creating a non-neoplastic cell-dominant tumor microenvironment.

There are only few studies in the literature regarding the significance of the number of HRS cells in CHL patients. In the past, nodular sclerosis CHL (NSCHL) was suggested to be graded per defined criteria, one of which was the number of neoplastic cells [3,4,5]. But then, some studies showed that grading does not have a significant effect on survival [6,7]. In more recent studies, a syncytial variant of NSCHL (SV-NSCHL), which is characterized by the presence of many HRS cell-forming sheets, has been associated with poor prognosis [8,9,10,11].

However, to the best of our knowledge, there is no study in the literature that investigates the prognostic effect of the number of neoplastic cells in CHL patients, regardless of the histological subgroup. Therefore, in this study, we aimed to evaluate whether the number, ratio, and/or grouping of HRS cells affect survival in patients with CHL. Considering almost all HRS cells express CD30, we used hematoxylin–eosin and immunohistochemically CD30-stained slides for the evaluation.

## 2. Materials and Methods

This study’s protocol was approved by the institutional ethics committee (2025/29-18).

### 2.1. Case Selection

A total of 135 consecutive adult patients who had been diagnosed with CHL in 117 excisional and 12 incisional biopsies were included in this study. Clinical and histopathological features were obtained from the hospital records.

### 2.2. Evaluation of HRS Cells

A demonstrative hematoxylin–eosin (HE)-stained slide and CD30 (anti-CD30 monoclonal antibody, Roche, Ventana, AZ, USA)-stained slide were re-reviewed by a pathologist (B.P.) (with more than ten years of experience in pathology) to identify the high-power field (×400 magnification) with the highest number of HRS cells. Areas with the highest number of neoplastic cells were marked using the eye-balling method (B.P). These selected “hot-spot” areas (both on HE- and CD30-stained slides) were photographed (by N.T.), and the number of HRS cells were counted. One photo representing a high-power field (original magnification ×400) was used for each case. These photographs were also used to determine the ratio of HRS cells, and the ratio was scored as follows: 1—HRS ratio 1–5%, 2—HRS ratio 6–25%, 3—HRS ratio 26–50%, 4—HRS ratio 51–75%, and 5—HRS ratio > 75%.

**Figure 1:**
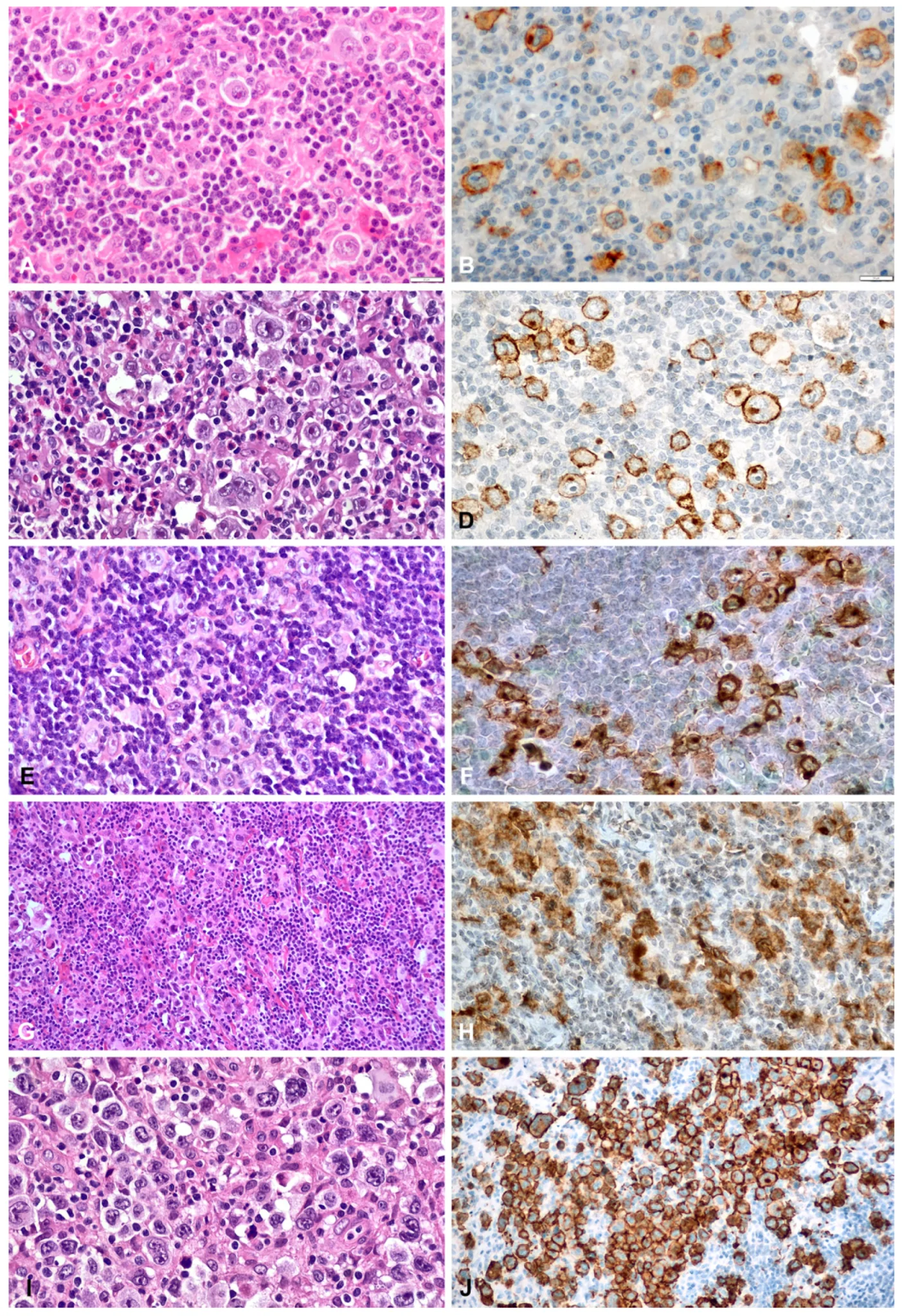
Scoring of HRS groups. (**A**,**B**) 0: No groups, (**C**,**D**) 1: 1–2 separate groups, (**E**,**F**) 2: 3–4 separate groups, (**G**,**H**) 3: ≥5 separate groups, and (**I**,**J**) 4: confluent groups and/or large groups of neoplastic cells. (**A**,**C**,**E**,**G**,**I**) Hematoxylin–eosin, original magnification ×400 and (**B**,**D**,**F**,**J**) CD30 immunohistochemistry, original magnification ×400.

The HE- and CD30-stained slides were also reviewed to evaluate the presence of HRS groups. An HRS group was defined as the clustering of ≥5 HRS cells in a high-power field. The cases were then scored according to the number of these groups: 0: no groups, 1: 1–2 separate groups, 2: 3–4 separate groups, 3: ≥5 separate groups, and 4: confluent and/or large groups of neoplastic cells (Figure 1).

The cut points were empirically chosen based on histopathological principles and the limits of rapid visual assessment to maximize inter-observer reproducibility.

For the HRS cell ratio, the initial tier in the range of 1–5% was selected to represent the biological baseline of CHL, where neoplastic cells typically constitute a very minor fraction (<5%) of the tumor microenvironment. The subsequent progressively widening intervals (6–25%, 26–50%, 51–75%, and >75%) were chosen to capture the gradual depletion of the stroma.

For the grouping scores, the discrete numerical cutoffs (1–2, 3–4, and ≥5 separate groups) were determined based on the ability of the human eye to instantly and accurately assess small numbers without tedious counting. Once the number of discrete groups reaches 5 or more (score 3), exact quantification becomes prone to inter-observer error. Score 4 (confluent/large groups) indicates the transition from localized clustering to solid, aggressive, sheet-like growth patterns.

### 2.3. Statistical Analysis

Statistical analyses were performed using the open software Jamovi project (https://www.jamovi.org/, Version 2.6.45). Descriptive analyses were performed. A chi-square test was used to compare categorical variables and the Kruskal–Wallis test was performed to compare two or more independent sample groups based on normality test results. Overall survival (OS) was defined as the time period from the date of biopsy until death from any cause or last follow-up date for surviving patients. Survival curves were obtained using the Kaplan–Meier method. A log-rank test was performed to compare survival between ≥2 groups. Significant variables in the univariate analysis were used in multivariate analyses. To identify independent prognostic factors for OS, a multivariable Cox proportional hazards regression model was utilized. The model-building strategy involved a two-step approach. First, univariate analysis was performed, and covariates that demonstrated a potential association with the outcome (*p* < 0.05) were selected as candidates. These variables were then entered into the multivariable model using a backward stepwise (Wald) elimination method to adjust for confounders. Results are expressed as hazard ratios (HRs) with corresponding 95% confidence intervals (CIs). *p* < 0.05 was considered statistically significant for all analyses.

## 3. Results

### 3.1. Clinicopathological Characteristics

Female:male ratio was 0.82 and the majority of the patients were male (*n* = 74). The median age was 36 ± 15.8 (range: 18–82 years). The most common location was cervical lymph nodes, followed by supraclavicular (*n* = 25), axillary (*n* = 10), and inguinal (*n* = 9) lymph nodes. Only one patient (0.8%) had stage I disease, and 58 (43.9%), 33 (25%), and 40 (30.3%) had stage 2, 3, and 4 diseases, respectively. While the majority (71.5%, *n* = 93) had NSCHL, 31 (23.8%) had mixed-cellularity, 5 (3.8%) had lymphocyte-rich, and the remaining one patient (0.8%) had lymphocyte-depleted CHL. Of 93 cases with NSCHL, 46 had SV-NSCHL. Epstein–Barr Virus-Encoded Small-RNA (EBER) positivity was detected in 48 patients (36.6%).

### 3.2. Results of Evaluation of HRS Cells

A Hodgkin cell was described as large or giant cell with a single large nucleus and prominent, eosinophilic inclusion-like nucleoli, and a Reed–Sternberg cell was described as a large cell containing a bilobed nucleus with inclusion-like nucleoli creating the appearance of an owl’s eye. Large cells with similar multiple nuclei were also considered to be neoplastic. The median numbers of HRS cells on HE- and CD30-stained slides were 32 ± 18.5 (range: 7–105) and 40 ± 27.8 (range: 9–146), respectively. The number of HRS cells on HE- and CD30-stained slides were strongly correlated (Spearman’s rho = 0.69, *p* < 0.001). No significant correlation between the number of HRS cells and age was observed. There was no significant association between gender and the number of HRS cells. The mean number of HRS cells did not significantly differ between early-stage (stages I–II) and advanced-stage (stages III–IV) patients (Table 1). The number of HRS cells on HE- and CD30-stained slides were significantly higher in NSCHL than in mixed-cellularity CHL and lymphocyte-rich CHL (mean 37.7 vs. 26.6 and 15.2, *p* = 0.005 and 0.011, and mean 50.4 vs. 35 and 18.2, *p* = 0.019 and 0.015, respectively) (Table 1). Also, the number of HRS cells was significantly higher in SV-NSCHL patients (mean 43.7 vs. 29.8 in HE and 60.2 vs. 38.5 in CD30-stained slides, *p* < 0.001). There was no significant association between the number of HRS cells and EBER positivity. However, EBER positivity was significantly more common in the mixed-cellular subtype compared to NSCHL (*p* < 0.001).

HRS cell ratio was in the range of 6–25% (score 2) in more than half of the cases (Table 2). There was no significant association between HRS ratio and gender, age, or stage. EBER positivity was more common among cases with a score 1 HRS ratio in HE-stained slides (*p* = 0.03). HRS cell ratio significantly differed between nodular sclerosis, mixed-cellular, and lymphocyte-rich subtypes (Table 2).

Of 135 cases, 49 had confluent and/or large groups of HRS on HE slides and 64 on CD30-stained slides (Table 3). The number of cases with a group score of 0 (no HRS groups) was significantly higher among men compared to women (37 vs. 15, *p* = 0.049 for HE and 28 vs. 13 for CD30, *p* = 0.012). The HRS group with score 0 was significantly more common in patients ≥40 years of age (54.9% vs. 28.6%, *p* = 0.007 for HE and 49% vs. 19.3%, *p* = 0.005 for CD30). No significant association was found between HRS group score and stage. All 5 patients with lymphocyte-rich CHL had a HRS group score of 0, i.e., no HRS group was present in these cases (*p* < 0.001). Confluent/large HRS groups were more common in EBER-negative patients while HRS cells mostly did not form groups in EBER-positive patients (*p* < 0.001). Confluent/large groups were also more common in SV-NSCHL cases (30/46 vs. 17/84 for HE and 40/46 vs. 22/83 for CD30, *p* < 0.001).

### 3.3. Survival Analysis Results

Median follow-up time was 50.2 months, follow-up was complete for 95.5% of the cohort, and we observed 6 relapses and 16 deaths during this period.

Mean OS time was 114 ± 3.74 months and the 5-year OS rate was 88.8%. A complete response could have been achieved in 119 (88.1%) cases. Partial response was observed in 2 cases (1.48%) while the remaining 8 patients (5.92%) did not respond to therapy. Response to first-line treatment was observed in 108 patients and was significantly associated with longer mean OS (120.8 ± 3.18 vs. 87.8 ± 11.63 months, *p* = 0.0002). OS was significantly longer in stage I–II disease (mean OS 126 ± 3.30 vs. 103 ± 5.97 months for stages I–II vs. stage III–IV, *p* = 0.014). OS was significantly shorter in patients ≥40 years of age (102 ± 6.85 vs. 122 ± 3.93 months, *p* = 0.0051). Also, mean OS was significantly shorter in males than in females (105 ± 6.02 vs. 123 ± 3.91 months, *p* = 0.0093). In 13 patients, brentuximab was used, and while the mean OS was shorter in patients who underwent brentuximab therapy (91.9 ± 15.13 vs. 117.1 ± 3.56 months, *p* = 0.027), response to brentuximab therapy was not associated with OS (88.1 ± 0 vs. 63 ± 9.07 months, *p* = 0.22). EBER positivity was associated with significantly shorter OS (mean OS 99.2 vs. 122.7 ± 3.34 months *p* = 0.0015). The effects of response to first-line therapy (HR 0.22, 95% 0.06–0.28, *p* = 0.02), gender (HR 5.51, 95% 1.09–27.80, *p* = 0.039), and EBER positivity (HR 0.24, 95% 0.06–0.99, *p* = 0.049) on OS persisted when these six factors were modeled together. The number, ratio, and grouping of HRS cells were not associated with OS (*p* > 0.05). Although patients with SV-NSCHL tended to live longer, the difference was not statistically significant (124 ± 3.7 vs. 111 ± 5.02 months, *p* = 0.096).

Response to first-line therapy was significantly more common in stage I–II cases compared to stage III–IV cases (54/59 vs. 52/72, *p* = 0.01). The mean number of HRS cells was lower in cases who responded to first-line therapy but difference between the groups was not statistically significant (*p* > 0.05). No other association was found regarding response to first-line therapy.

Recurrence occurred in 6 (4.44%) patients and the mean time to recurrence was 36 ± 13.6 months (range: 18.8–57.1 months). However, the presence of recurrence was not associated with OS (*p* = 0.43). No significant association was found between recurrence and age or gender. In 5 of the 6 patients with recurrence, there was no response to first-line therapy (*p* < 0.001). Recurrence was most common in patients with a group score of 3 in CD30-stained slides, however there was no significant association in pairwise comparisons between groups. No association was found between recurrence and other parameters.

## 4. Discussion

In this study of 135 consecutive cases with CHL, we investigated whether the number, ratio, and/or grouping of HRS cells affect prognosis, and we obtained some interesting results, which are discussed below.

HRS cell ratio was ≤25% in the majority of cases, as expected. The number of HRS cells and HRS cell ratio significantly differed among CHL subtypes, with NSCHL (particularly the syncytial variant) having significantly more HRS cells. All five patients with lymphocyte-rich CHL lacked HRS groups. Confluent/large groups were more common in SV-NSCHL cases. These differences may be attributable to the different immune signatures in subtypes of CHL [12]. While they may be used as clues for subtyping in addition to the background characteristics during diagnostic evaluation, the number, ratio, and/or grouping of HRS cells did not have any direct prognostic implication as they were not associated with stage, OS, response to first-line therapy, and/or recurrence. In the past, mean tumor burden normalized to body surface area on computed tomography has been reported to be a prognostic factor for CHL [13]. Therefore, the dissemination of the disease seems to be more important than the volume of neoplastic cells in CHL. Miljak et al. [14] recently claimed that high PD-L1 expression in HRS cells and macrophages in the tumor immune microenvironment is associated with shorter survival in CHL cases. Zargari et al. [15] showed that a lack of immunohistochemical STAT1 or pSTAT3 expression in HRS cells is associated with poor prognosis. A macrophage-like genomic expression in CHL cases has been found to be associated with treatment failure [16]. Also, in a cell culture study, small and large HRS cells have been shown to have genomic similarities and differences [17]. When our findings are interpreted alongside these studies, it is reasonable to say the quality matters more than quantity in regard to HRS cells in CHL cases, particularly when HRS cells have the ability to govern inflammatory cells in the vicinity via immune pathways. There are also several studies on the effect of the microenvironment on the behavior of CHL [18,19,20]. Our results also raise the question of whether the prognostic effect of the CHL microenvironment may be more important than that of HRS cells alone.

Our findings also challenge the previous reports suggesting that SV-NSCHL, which is characterized by an abundance and cohesion of HRS cells, exhibits poorer prognosis [8,9,10,11]. In fact, our analysis revealed that patients with SV-NSCHL tended to live longer. This may be attributable to a lack of any significant association between the presence of SV-NSCHL and any aggressive disease indicator, such as no response to first-line therapy, recurrence, and/or being at an advanced stage in our study group. On the other hand in a previous study, two of the three pediatric cases with SV-NSCHL showed complete response to therapy [21]. In our study group, complete response was achieved in 43 out of 46 cases with SV-NSCHL. Therefore, it is possible to achieve complete response in SV-NSCHL cases. It should also be noted here that our study design is retrospective, the treatment regimen was not controlled, and there were differences in treatment modules, although they were based on an ABVD (doxorubicin, bleomycin, vinblastine, and dacarbazine) regimen (cycles changing between 2 and 8). While 67 patients received only ABVD, 41 underwent radiotherapy in addition to ABVD, bone marrow transplantation was performed on 16 patients, and 5 had nivolumab therapy. Thus, we think that response to first-line therapy, presence of recurrence, and/or being at an advanced stage are more objective response measures in our study, and SV-NSCHL was not associated with any of these factors, supporting our finding that the number and/or grouping of HRS cells (even forming SV-NSCHL) is not a direct prognostic factor alone. Also, populational differences may be at play, and therefore, multi-institutional prospective studies may help to explain these different results.

Although the number of HRS cells does not seem to have a direct prognostic effect, we observed some minor findings that are pertinent to independent prognostic factors. Response to first-line therapy, gender, and EBER positivity were found to be independent prognostic factors both in univariate and multivariate analyses.

Response to first-line therapy was significantly associated with longer mean OS. The mean number of HRS cells was lower in cases that responded to first-line therapy, albeit without statistical significance. This finding should be interpreted cautiously considering statistical insignificance. However, we think that this finding should be verified in larger study groups as it may have an impact on the type and/or length of first-line treatment.

We observed significantly shorter OS in males compared to females. Male sex has already been described as a risk factor for advanced-stage CHL [22] and longer survival times have been observed for females with CHL [23,24,25]. Survival advantage of women is thought to be a result of estrogen production [26]. Nevertheless, ERβ activation has been shown to result in the inhibition of cell proliferation and cell cycle progression by inducing autophagy in Hodgkin lymphoma patients [27]. Curiously, in our study, HRS group formation was less frequent among men compared to women. Estrogens have been demonstrated to promote cell–cell adhesion in normal and cancerous breast cells [28], and we think that a similar mechanism may be responsible for the lack of HRS groups in male patients.

In our study, EBER positivity was associated with significantly shorter OS, which is in agreement with previous studies [29]. While confluent/large HRS groups were more common in EBER-negative patients, HRS cells usually did not form groups in EBER-positive patients. This may be a reflection of the lower frequency of EBER positivity in NSCHL patients. The number of HRS cells was lower in EBER-positive cases, but the difference was not statistically significant, similar to a previous study [30]. The lack of HRS group formation in EBER-positive cases may be attributed to the down-regulation of cell adhesion molecules such as CD99 [31,32,33]; however, this hypothesis fails to explain frequent group formation in EBER-negative cases. Moreover, the tumor microenvironment possibly differs in EBER-positive and -negative patients. Further investigation is needed to explore this aspect.

OS was significantly shorter in patients ≥40 years of age (102 ± 6.85 vs. 122 ± 3.93 months, *p* = 0.0051). CHL in older patients causes poorer survival rates [22,34,35,36], probably due to comorbidities and the toxic effects of the treatment. In our study, more than two thirds of patients ≥40 years had advanced-stage disease, which may explain the survival disadvantage. Also, a HRS group score of 0 was significantly more common in patients ≥40 years of age, most likely a reflection of male predominance in this group.

Our study has some limitations. First, our study group consisted predominantly of patients with NSCHL, and there were only five cases of lymphocyte-rich and one case of lymphocyte depleted CHL. While the predominance of nodular sclerosis type is expected, lymphocyte-rich and lymphocyte-depleted subtypes could not be represented adequately. Sample size may have also affected the results in subgroup analyses. Secondly, it may be challenging to count Hodgkin cells on HE due to fixation artifacts. To minimize the effect of such situations, we evaluated the HRS cells on CD30-stained slides as well, which we believe was useful to overcome this limitation. Moreover, the selected areas represented a very small percentage of the entire biopsy specimen; therefore, it is not possible to make extrapolations regarding the effect of neoplastic cell burden on prognosis. Interobserver agreement was not analyzed, and this limits the reproducibility. Also, we did not use digital image analysis for cell counting. While some schools may claim that digital image analysis provides precise numbers, we think that our manual counting method reflects the real-world daily practice of a surgical pathologist. Another aspect is the heterogeneous treatment modalities within the study group due to the retrospective design. While we think that this does not diminish the value of our findings, a prospective study design may provide more objective data regarding the treatment effect on prognosis. Lastly, disease-specific survival could not be estimated due to the limited data.

## 5. Conclusions

In conclusion, our results show that the number, ratio, and/or grouping of HRS cells do not have any significant prognostic impact on patients with CHL. However, future studies are required to explore the reasons for the differences between CHL subtypes, the mechanism of HRS cell grouping, and its effects on pathogenesis.

## Figures and Tables

**Table 1:** Associations between the number of HRS cells and clinicopathological parameters.

	Mean # of HRS Cells			
	HE-Stained Slides	*p*	CD30-Stained Slides	*p*
**Gender**				
**Male (*n* = 74)**	34.2 ± 21.1 (Range 7–105)	0.87	43.7 ± 29 (Range 9–146)	0.29
**Female (*n* = 61)**	34.7 ± 15 (Range 10–77)		48.7 ± 26.1 (Range 9–116)	
**Age**				
**<40 (*n* = 84)**	35.3 ± 16.4 (Range 8–91)	0.45	47.5 ± 25.4 (Range 13–144)	0.41
**≥40 (*n* = 51)**	32.9 ± 21.7 (Range 7–105)		43.4 ± 31.4 (Range 9–146)	
**Subtype**				
**Nodular sclerosis (*n* = 93)**	37.7 ± 17.6 (Range 11–91)	**<0.001**	50.4 ± 27.5 (Range 14–144)	**<0.001**
**Mixed cellularity (*n* = 31)**	26.6 ± 15.2 (Range 7–82)		35 ± 20.3 (Range 9–106)	
**Lymphocyte-rich (*n* = 5)**	15.2 ± 7.8 (Range 8–28)		18.2 ± 9.2 (Range 9–30)	
**Lymphocyte-depleted (*n* = 1)**	105		146	
**SV-NSCHL (*n* = 46)**	43.7 ± 17.3 (Range 12–79)		60.2 ± 24.5 (Range 14–116)	
**Non-SV-NSCHL (*n* = 84)**	29.8 ± 17.7 (Range 7–105)	**<0.001**	38.5 ± 27.1 (Range 9–146)	**<0.001**
**EBER status**				
**Positive (*n* = 48)**	31 ± 21.2 (Range 7–105)	0.07	43.1± 30.4 (Range 9–146)	0.28
**Negative (*n* = 83)**	37.1 ± 16.6 (Range 12–91)		48.6 ± 26.2 (Range 13–144)	
**Stage**				
**Stages I–II**	32.9 ± 16.8 (Range 7–91)	0.49	44.9 ± 26.8 (Range 9–144)	0.72
**Stages III–IV**	35.2 ± 19.9 (Range 8–105)		46.6 ± 28.9 (Range 9–146)	

**Table 2:** Associations between HRS cell ratio and clinicopathological parameters.

		Subtype				*p*	SV Status		*p*
		Nodular Sclerosis	Mixed Cellularity	Lymphocyte-Rich	Lymphocyte Depleted		Non-SV-NSCHL	SV-NSCHL	
**HE**	**1 (*n* = 24)**	9 (39.1%)	9 (39.1%)	5 (21.7%)	0 (0.0%)	**<0.001**	21 (91.3%)	2 (8.7%)	**<0.001**
	**2 (*n* = 76)**	55 (74.3%)	19 (25.7%)	0 (0.0%)	0 (0.0%)		48 (64.9%)	26 (35.1%)	
	**3 (*n* = 29)**	23 (85.2%)	3 (11.1%)	0 (0.0%)	1 (3.7%)		15 (55.6%)	12 (44.4%)	
	**4 (*n* = 5)**	5 (100.0%)	0 (0.0%)	0 (0.0%)	0 (0.0%)		0 (0.0%)	5 (100.0%)	
	**5 (*n* = 1)**	1 (100.0%)	0 (0.0%)	0 (0.0%)	0 (0.0%)		0(0.0%)	1 (100.0%)	
**CD30**	**1 (*n* = 27)**	12 (46.2%)	9 (34.6%)	5 (19.2%)	0 (0.0%)	**<0.001**	24 (92.3%)	2 (7.7%)	**<0.001**
	**2 (*n* = 72)**	49 (71.0%)	20 (29%)	0 (0.0%)	0 (0.0%)		48 (69.6%)	21 (30.4%)	
	**3 (*n* = 29)**	26 (92.9%)	2 (7.1%)	0 (0.0%)	0 (0.0%)		11 (39.3%)	17 (60.7%)	
	**4 (*n* = 3)**	3 (100.0%)	0 (0.0%)	0 (0.0%)	0 (0.0%)		0 (0.0%)	3 (100.0%)	
	**5 (*n* = 4)**	3 (75.0%)	0 (0.0%)	0 (0.0%)	1 (25.0%)		1 (25.0%)	3 (75.0%)	

HRS cell ratios were scored as follows: 1—HRS ratio 1–5%, 2—HRS ratio 6–25%, 3—HRS ratio 26–50%, 4—HRS ratio 51–75%, and 5—HRS ratio > 75%. SV: Syncytial variant.

**Table 3:** Distribution of HRS groups.

	Group	# of the Cases
**HE-stained slides**	**0**	52
	**1**	18
	**2**	8
	**3**	8
	**4**	49
**CD30-stained slides**	**0**	41
	**1**	14
	**2**	8
	**3**	7
	**4**	64

## Data Availability

Data are available upon reasonable request from the authors.
